# Antioxidant Activity of Pomegranate Husk Ellagitannins in Enhancing Oxidative Stability of Canola Oil During Frying

**DOI:** 10.3390/foods14020226

**Published:** 2025-01-13

**Authors:** Mariela R. Michel, Maritza Pacheco-Lara, Romeo Rojas, Guillermo Cristian G. Martínez-Ávila, Juan Alberto Ascacio-Valdés, Mayra Aguilar-Zárate, Pedro Aguilar-Zárate

**Affiliations:** 1 Laboratorio Nacional CONAHCYT de Apoyo a la Evaluación de Productos Bióticos-LaNAEPBi, Unidad de Servicio Tecnológico Nacional de México, Instituto Tecnológico de Ciudad Valles, Ciudad Valles 79010, San Luis Potosí, Mexico; mariela.michel@tecvalles.mx (M.R.M.); 18690372@tecvalles.mx (M.P.-L.); 2 Laboratory of Chemistry and Biochemistry, School of Agronomy, Autonomous University of Nuevo Leon, Francisco Villa S/N, Ex Hacienda El Canadá, General Escobedo 66050, Nuevo Leon, Mexico; romeo.rojasmln@uanl.edu.mx (R.R.); guillermo.martinezavl@uanl.edu.mx (G.C.G.M.-Á.); 3 Bioprocesses Research Group, Food Research Department, School of Chemistry, Universidad Autónoma de Coahuila, Saltillo 25280, Coahuila, Mexico; alberto_ascaciovaldes@uadec.edu.mx; 4 Facultad de Ciencias Químicas-CIEP, Universidad Autónoma de San Luis Potosí, Av. Dr. Manuel Nava 6, Zona Universitaria, San Luis Potosí 78210, San Luis Potosí, Mexico

**Keywords:** pomegranate husk polyphenols, ellagitannins, vegetable canola oil, frying, peroxides

## Abstract

This study evaluated the antioxidant efficacy of ellagitannins from a pomegranate husk in preventing vegetable canola oil (VCO) oxidation during French fry preparation. Ellagitannins were extracted using 80% acetone, purified via Amberlite XAD-16 resin chromatography, and incorporated into VCO at 0.05%, 0.1%, and 0.2% concentrations. VCO oxidation was assessed at 145 °C, 160 °C, and 190 °C, with frying experiments conducted at 160 °C for five 10 min cycles. Primary lipid oxidation (peroxide values) was measured using the AOCS Cd 8-53 method, and molecular structural changes were analyzed by infrared spectroscopy. Results showed that ellagitannins significantly mitigated VCO oxidation across all temperatures, with 0.05% identified as the optimal concentration. This concentration reduced peroxide values to 4.66 ± 1.15 meq O/kg, remaining stable and below acceptable limits during frying. Infrared spectroscopy confirmed no significant structural changes in VCO. These findings highlight ellagitannins as effective antioxidants for enhancing VCO oxidative stability during frying, offering a natural, sustainable solution for improving oil quality and extending its usability in the food industry.

## 1. Introduction

Edible vegetable oils are mainly composed of triacylglycerides with a high percentage of unsaturated fatty acids (95–99%) [1]. The higher degree of unsaturation in a fatty acid, that is, the more double bonds are present in the fatty acid, the more vulnerable it is to lipid oxidation [2]. This phenomenon in oils originates because the oxygen present in the environment reacts on the contact surface and attacks the double bonds and consequently a series of undesirable reactions begin to occur that produce unpleasant odors. Furthermore, oxidation can also be induced by high temperature, humidity, light, or by the presence of oxidizing metal ions such as Cu and Fe. Due to these reactions, a free radical is generated acting as an initiator of a chain of reactions that generate more free radicals and that, upon coming into contact with atmospheric oxygen, form peroxides or hydroxyperoxides, i.e., primary oxidation compounds [3,4,5]. These reactions are followed by others in which the propagation of peroxides takes place and ends with the formation of secondary compounds (aldehydes, ketones, and polymers, among others). Another common factor that causes the oxidation of edible oils is their overheating during food preparation, mainly at a commercial scale, which affects their nutritional composition (there is a loss of essential fatty acids and vitamins), as well as their organoleptic characteristics, changing their texture, smell, and color [6,7]. In addition, repeated heating of oil produces the formation of free radicals and in some cases trans fatty acids are generated. It has been seen that excessive and prolonged intake of oils with the presence of free radicals causes liver and heart dysfunction, has carcinogenic effects, increases cellular aging, prevents the absorption of nutrients necessary for the body, decreases good cholesterol (HLD), and increases bad cholesterol (LDL) [8]. Various antioxidant compounds have been identified that can inhibit the oxidation of vegetable oils, whose mechanism of action is based on the ability of their hydroxyl groups to capture free radicals, thus delaying the oxidative degradation of lipids and improving the quality and nutritional value of foods. Within the food industry, there is a wide variety of antioxidants, of synthetic and natural origin [9]. According to their mechanism of action, antioxidants can be primary, which are free radical terminators that delay the initiation stage or stop the propagation of autoxidation, e.g., butylated hydroxyanisole (BHA), butylated hydroxytoluene (BHT), propyl gallate, and tertiary butylhydroquinone (TBHQ). On the other hand, there are secondary antioxidants which reduce the rate of oxidation reactions by functioning as chelators for pro-oxidant metal ions, providing hydrogen to primary antioxidants, decomposing hydroperoxides to non-radical species, neutralizing singlet oxygen or acting as oxygen scavengers (e.g., ascorbic acid, ascorbic palmitate, carotenoids, and citric acid, among others) [10]. Most synthetic antioxidants have been reported to have mutagenic, teratogenic, and carcinogenic effects when present in high concentrations and/or if ingested over a long period of time [11]. For this reason, international regulatory institutions have established permissible limits for their use in edible vegetable oils, and these quantities are not always adequate to completely counteract the effects of oxidation. In recent years, some alternatives to natural antioxidants have been proposed, such as carotenoids, vitamins, and some phenolic compounds, that can replace synthetic antioxidants [12]. In this context, there has been an increase in interest in phenolic compounds extracted from agro-industrial by-products, due to their nutraceutical properties and because they effectively inhibit thermo-oxidative degradation, as well as the formation of toxic compounds such as acrylamide, peroxides, and aldehydes, among others [13] in products and processes where the addition of vegetable oils is required. In particular, it has been seen that polyphenols obtained from pomegranate peel (*Punica granatum)*, such as punicalagin, and ellagic acid, among others, present high antioxidant activity [14]. In addition, it has been shown that pomegranate peel extract can maintain up to 66% of its antioxidant activity after subjecting it to a heat treatment of 180 °C for 80 min [15]. This has led to proposals for the use of these extracts as a source of natural antioxidants to replace synthetic ones, for example, in sunflower oil that was used to fry chicken nuggets. Such work concluded that with the use of 500 to 700 ppm of extract, it was possible to stabilize the formation of free radicals present in the frying process [13]. This effect was mostly attributed to punicalagin, a polyphenol from the ellagitannin group, whose molecule has 16 dissociable -OH groups [16]. These chemical properties of punicalagin have allowed its application as an antioxidant in olive oil, where it significantly reduced the formation of peroxides (18.17 meq/kg of oil), compared to the synthetic antioxidant BHT, thus demonstrating that punicalagin may be able to improve the quality of the oil during storage and prolong its shelf life [17]. For the above reasons, it was considered that the application of pomegranate husk extract, rich in polyphenols too, could prolong the shelf life, during different heating cycles, and quality of oil used for frying foods. In this context, the aim of the present work was to evaluate the antioxidant effect of pomegranate husk polyphenols in canola vegetable oil used for a food frying process.

## 2. Materials and Methods

The pomegranate (*Punica granatum*), Valencia variety, to obtain the extract were purchased at the local farmer market of Ciudad Valles, San Luis Potosí. The reagents used during the methodology were pure acetone (Jalmek brand, San Nicolás de los Garza, Mexico), 96% ethanol (CTR brand, Pune, India), starch (Mi Granero brand, Barge, Italy), Amberlite XAD-16 (Sigma Aldrich brand, St. Louis, MO, USA), chloroform (Jalmek brand), acetic acid (Jalmek brand), potassium iodide, and sodium thiosulfate (Fagalab brand, Mocorito, Sinaloa, Mexico). Edible canola vegetable oil, CVO (Capullo brand, Rzeszow, Poland), was used for frying. Frozen cut potatoes of the McCain™ brand (Toronto, ON, Canada) were obtained from a local supermarket and used.

### 2.1. Drying of Pomegranate Husk

The pomegranate husk was removed from the fruit and separated. It was washed with water only and dried at 60 °C for 48 h in an oven (MEMMERT brand, Schwabach, Germany). The dried husks were ground in a manual mill (ESTRELLA brand, Burbank, CA, USA) until a particle size between 800 μm and 1200 μm was obtained.

### 2.2. Extraction of Polyphenols from Pomegranate Husk

The extraction and purification were carried out from dehydrated and pulverized pomegranate husk residues. Considering a 1:10 ratio, 80 g of pulverized sample were placed in a container and 800 mL of 80% acetone were added. Acetone was selected as the extraction solvent because it enables the recovery of higher quantities of hydrophobic ellagitannins. The extraction process consisted of taking the solid/solvent for 10 min to ultrasound (model 3800, BRANSON brand, Brookfield, CT, USA), after which the obtained extract was vacuum filtered using coffee filter paper to remove the largest residue particles and a second filtering was carried out with filter paper (Whatman No. 1) to remove the suspended particles. Once the aqueous extract was filtered, it was taken to the rotary evaporator (R-100, BUCHI brand, Mumbai, India) to remove the solvent, obtain the extract, and proceed to purification.

### 2.3. Purification of Polyphenols

The purification process was conducted in accordance with the methodology reported by Ascacio-Valdés et al. [18]. A column (Kimble-flex 2.5 cm × 75 cm, 344 mL) filled with 100 g of Amberlite XAD-16 was employed to separate the compounds of interest on the basis of their differing polarities, with non-polar compounds (such as carbohydrates) being separated from the glucosides present in most plant extracts, which otherwise hinder the concentration of phenolic compounds. At the outset of the treatment, the column was first washed with ethanol and then with distilled water. The purification was conducted by placing 50 mL of extract and a preliminary wash with water to eliminate undesired compounds, followed by the addition of ethanol to obtain a fraction enriched in ellagitannins. At this juncture, the sample was retrieved. This process was repeated until the desired volume was obtained. The purified sample was subsequently subjected to rotary evaporation (R-100, BUCHI) in order to remove the majority of the ethanol. The concentrate was then transferred to Petri dishes and dried in an oven at 50 °C for 48 h. Finally, the sample was recovered by scraping the surface of the Petri dish, and the resulting powder was stored in Eppendorf tubes covered with aluminum foil in a cool, dry place until the next step.

### 2.4. HPLC-ESI-MS/MS Characterization

The ethanolic fractions (2000 ppm) were analyzed via reversed phase high performance liquid chromatography (RP-HPLC) in tandem with mass spectrometry according to Aguilar-Zárate et al. [19]. The RP-HPLC includes an auto-sampler (Varian ProStar 410, Palo Alto, CA, USA), a ternary pump (Varian ProStar 230I), and a photodiode array (PDA) detector (Varian ProStar 330). Samples (10 μL) were injected onto a Denali C18 column (150 mm × 4.6 mm, 3.1 μm, Grace, Columbia, MD, USA). The oven temperature was 30 °C. The mobile phase was composed of aqueous formic acid (0.2%, *v*/*v*; solvent A) and acetonitrile (solvent B). The solvents and samples were filtered through 0.45 μm nylon membranes. The flow rate was set at 0.2 mL/min, and elution of phenolic compounds was monitored at 280 and 375 nm. Data processing was performed by Workstation Multi Instrument (V. 6.2). The gradient applied was as follows: initial, 3% B; 0–5 min, 9% linear B; 5–15 min, 16% linear B; 15–45 min, 50% linear B. The column was then washed and reconditioned.

A liquid chromatograph ion trap mass spectrometer (Varian 500-MS IT Mass Spectrometer, Agilent Technologies, Santa Clara, CA, USA) equipped with an electrospray ion source was used. The samples were submitted in tandem from the HPLC to the ESI-MS. All MS experiments were carried out in the negative mode [M-H]^−1^. Nitrogen was used as nebulizing gas and helium as damping gas. The ion source parameters were spray voltage 5.0 kV, and capillary voltage and temperature were 90.0 V and 350 °C, respectively. Data were collected and processed using MS Workstation software (V 6.9). Full scan spectra were acquired in the *m*/*z* range 50–2000. Samples were firstly analyzed in full scan mode. MS/MS analyses were performed on a series of selected precursor ions.

### 2.5. Evaluation of the Antioxidant Effect of Polyphenols in Canola Vegetable Oil

The evaluation of the functionality of polyphenols as antioxidants in CVO was carried out based on the considerations reported by FAO, which indicates that the temperature range that frying oil for snack-type products should reach is between 140 °C and 200 °C. The polyphenols were added to canola vegetable oil prior to heat treatment. For this experimental design, 10 g of oil samples in Erlenmeyer flasks were considered, without polyphenols (0%) and with polyphenols 0.05%, 0.1%, or 0.2% (concentrations previously established according to the limits of NMXF-808-SCFI-2018 [20] for the use of antioxidants in edible vegetable oils) and three levels of frying temperatures were established (i.e., 145 °C, 160 °C, and 190 °C). All heat treatments were carried out in an electric fryer (model WM-16116, FARBERWARE brand, New York, NY, USA) for 90 min. After each procedure was completed, the sample was allowed to cool to room temperature for further use.

### 2.6. Application of Polyphenols to Vegetable Oil for Frying Potatoes

Based on the conditions obtained in the statistical analysis of the results of the preliminary process, it was decided to add 0.1% of polyphenols to the vegetable oil for frying. It is worth mentioning that 0.1% was the intermediate concentration of polyphenols with which a significant inhibition of oxidation could be achieved (*p* < 0.05), keeping the peroxide levels within the limit established by the standards (10 mEq O_2_/kg; CFR-Code of Federal Regulations Title 21, U.S. FDA [21]). For the pre-frying process, one liter of vegetable oil was added to the tank of the electric fryer and brought to 160 °C. Once the temperature was reached, 300 g of frozen cut potatoes were added and fried for 10 min. At the end of this time, an aliquot of oil was taken and labeled with cycle 1 and so on until the 5 frying cycles were completed, using a new batch of potatoes in each cycle. The aliquots were cooled to room temperature and stored in amber glass jars until use. The control treatment was processed in the same way using oil without polyphenols.

### 2.7. Evaluation of Primary Lipid Oxidation

Lipid oxidation in canola oil was determined using the American Oil Chemists’ Society (AOCS) acetic acid-chloroform method for quantification of peroxide values (AOCS official method Cd 8-53) [22]. Briefly, 5 g of the sample was dissolved in 30 mL of a 3:2 mixture of glacial acetic acid and chloroform in a glass-stoppered Erlenmeyer flask. Subsequently, 0.5 mL of a saturated potassium iodide solution was added, and the mixture was allowed to react in the dark for 1 min. Afterward, 30 mL of distilled water was added, and the liberated iodine was titrated with 0.01 N sodium thiosulfate solution until the yellow color almost disappeared. Then, 0.5 mL of freshly prepared starch indicator solution was added, and titration continued until the blue color disappeared. A blank determination was performed under identical conditions. All treatments were performed in triplicate. Results were reported in milliequivalents of peroxides present per kilogram of oil:

$$IP=\frac{\left(S-B\right)*N*1000\;}{\;m}=mEq/{kg}^{-1}$$

where *S* is the mL of sodium thiosulfate spent in the sample, *B* is the mL of sodium thiosulfate spent in the control, *N* is the normality of sodium thiosulfate, and *m* is the weight of the sample.

### 2.8. Identification of Peroxide Formation by Infrared Spectroscopy

To determine the changes in the functional groups of the fatty acids that make up the vegetable oil when polyphenols are added (i.e., oxidation inhibition), the samples from each heating cycle were analyzed in an Agilent Cary 630 (Agilent Technologies, Santa Clara, CA, USA) infrared spectrophotometer coupled to a zinc selenide (ZnSe) crystal ATR. A total of 32 scans were taken with a resolution of 2 cm^−1^ in the spectrum range from 4000 to 650 cm^−1^.

### 2.9. Statistical Analysis

Each sample was processed in triplicate and the data were statistically analyzed by ANOVA using Statistica v10 software. The analysis of the spectrum and the detected functional groups was performed with the MicroLab program, and the graph was constructed with the OriginPro 2018 program.

## 3. Results and Discussion

### 3.1. Polyphenols from Pomegranate Husk

The maximum polyphenol yield obtained from pomegranate husk was 13.75% (±2.0%) in *w*/*w* ratio, which was considerably higher than the 5% obtained by Martínez-Torres (2023) [23], from this same by-product. The above differences can be attributed to the affinity between the polyphenols and the solvent used to extract them (i.e., water) and additionally to the fact that in the present work two additional filtration processes were used to recover the extract.

**Figure 1:**
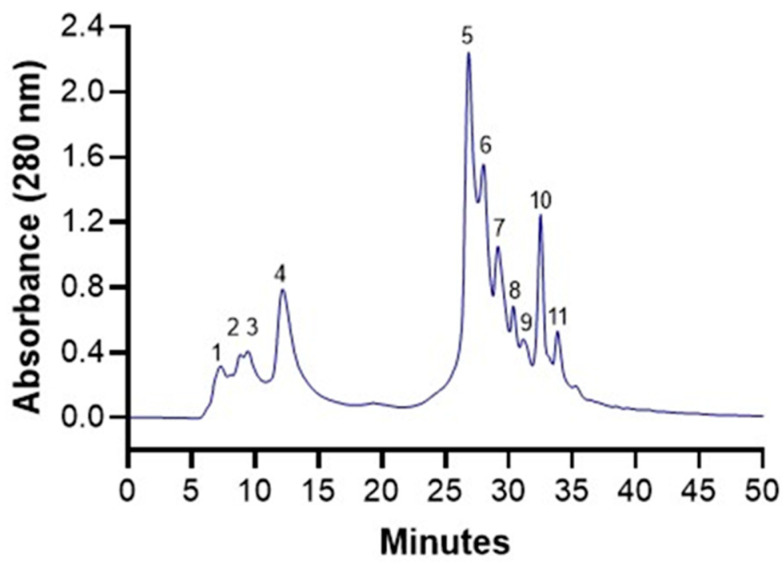
HPLC chromatogram for the phytochemicals purified from the pomegranate husk.

After the partial purification of polyphenols, an HPLC-ESI-MS analysis was carried out. A total of 11 phytochemicals were identified (Figure 1). Table 1 shows the tentative identification of the 11 phytochemicals based on retention time, maximal wavelength, mass-to-charge ratio, and MS^2^ fragmentation pattern. The main phytochemicals were the ellagitannins: punicalagin, punicalin, and granatin B. For this reason, the present work highlights that the antioxidant properties of pomegranate husk extract are mainly due to the ellagitannins it contains.

Previous studies have reported the presence of punicalagin anomers and ellagic acid derivatives in *Valenciana* pomegranate husk [21]. However, those studies employed water as the extraction solvent, whereas in the present study, aqueous acetone was used.

Table 1 provides the tentative identification of the phytochemicals from the pomegranate husk. Peak 1 was identified as galloyl hexoside based on the parent ion at *m*/*z* 331. The MS^2^ analysis corroborated this identification by showing a fragment at *m*/*z* 169, which corresponds to the loss of a hexose moiety, as previously reported [24]. Peak 2 exhibited a *m*/*z* of 707, indicative of a doubly charged ion. MS^2^ analysis revealed fragments at *m*/*z* 613 and 633, which, according to Mena et al. [25], identify the molecule as di(HHDP-galloyl glucose)-pentose (*m*/*z* 1415).

Peak 3 corresponded to pedunculagin I, with a parent ion at *m*/*z* 783. The MS^2^ fragments at *m*/*z* 481 and 301 indicated the loss of ellagic acid and the formation of HHDP-hexoside [18]. Punicalin (*m*/*z* 781) or gallagyl-hexoside was assigned to peak 4. This compound was identified based on the presence of a fragment at *m*/*z* 601, corresponding to the loss of a hexose group and formation of the gallagic acid moiety. Peak 5 was identified as punicalagin (*m*/*z* 1083), corroborated by the presence of fragments at *m*/*z* 781 (loss of ellagic acid) and *m*/*z* 601 (loss of the gallagic acid moiety) [26]. Notably, the β-anomer of punicalagin was not separated using the current HPLC method, likely due to limitations in chromatographic method resolution.

Identification of a compound with *m*/*z* 799 (peak 6) is unclear. Authors have mentioned that it could be granatin A or lagerstannins [18,23]. It is definitely an ellagic acid derivative due to the presence of fragments *m*/*z* 781, 479, and 301. Another compound with unclear identification is the one signaled as peak 7. Tentatively, it was identified as sanguiin H-6 since the doubly charged ion (*m*/*z* 934) indicates half the mass of the molecule (m.w. 1871.3 g/mol, *m*/*z* 1870) [27,28]. However, the fragment with *m*/*z* 801 does not help us to completely corroborate the identity of the molecule.

The peak 8 was identified as pedunculagin II or digalloyl-HHDP-hexoside. The molecule had a *m*/*z* oof 785 and a fragmentation pattern that corresponded to the release of digalloylhexoside (*m*/*z* 483), ellagic acid (*m*/*z* 301), and galloil-HHDP-hexoside (*m*/*z* 633). This later was also identified as peak 9 and has the following fragments: *m*/*z* 301, 435, 463.

Granatin B was identified with an [M-H]-ion at *m*/*z* 951. The MS^2^ analysis revealed the formation of ion *m*/*z* 301; that indicates the formation of ellagic acid since the compound has two HHDP groups attached to an hexose core [29]. Finally, the ellagic acid was identified with the *m*/*z* 301 and typical fragmentation pattern of *m*/*z* 301, 257, 272 [24].

### 3.2. Effect of Polyphenol Addition in Vegetable Canola Oil for Frying

**Figure 2:**
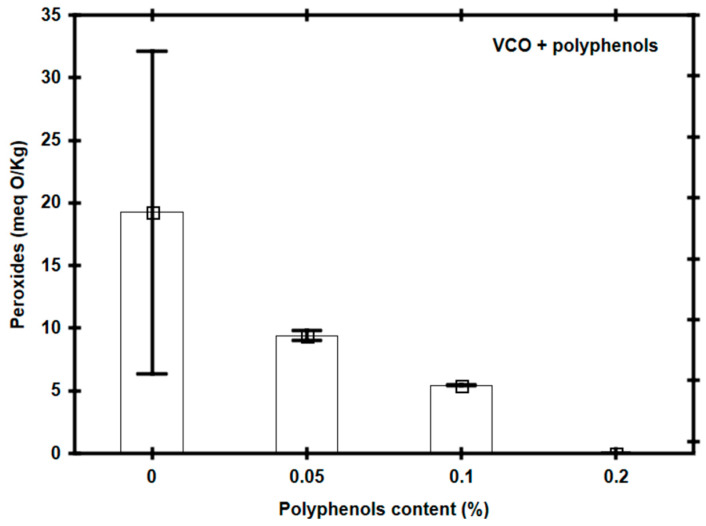
Peroxide concentration in vegetable canola oil (VCO) with different percentages of polyphenols, after having been heated at 160 °C for 90 min.

The functionality of three concentrations of pomegranate husk polyphenols (0.05%, 0.1%, and 0.2%) as antioxidants in vegetable canola oil (VCO), heated to 160 °C for 90 min, was preliminarily evaluated by determining the presence of peroxides as an indicator of primary oxidation of the oil. The results of this first experimental phase showed that the use of polyphenol concentrations of 0.1% or 0.2% partially or totally inhibited the peroxide formation in the VCO (Figure 2) after a heating treatment at 160 °C for 90 min (i.e., 0%: 19.25 meq O/Kg ± 6.44 meq O/Kg; 0.05%: 9.42 meq O/Kg ± 0.21 meq O/Kg; 0.1%: 5.43 meq O/Kg ± 0.01 meq O/Kg; 0.2%: 0 meq O/Kg ± 0.00 meq O/Kg). For this reason, in subsequent experiments of this work it was decided to use polyphenol concentrations of 0.1%, whose concentration implies the lowest expense, is within the limits established by international standards for the use of additives in food products [21] and with which it was possible to maintain the peroxide levels under the limits established by the standards (i.e., less than 10 mEq O/Kg).

**Figure 3:**
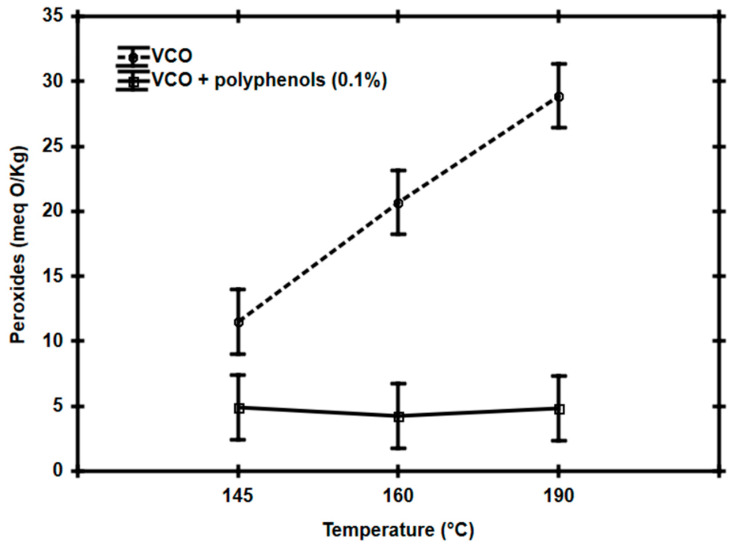
Peroxide formation in vegetable canola oil (VCO) without or with 0.1% pomegranate husk polyphenols, after heating at 145 °C, 160 °C, or 190 °C for 90 min.

The results of the inhibition of peroxide formation in frying oil by the addition of 0.1% of polyphenols, after being heated to 145 °C, 160 °C or 190 °C for 90 min, contrasting with the VCO samples without polyphenols under the same conditions, are shown in Figure 3. It was found that, in frying oil without polyphenols, the increase in peroxide concentration was directly proportional to the increase in heating temperature to which it was subjected (i.e., at 145 °C: 11.48 meq O/Kg (±3.11 meq O/Kg); at 160 °C: 20.68 meq O/Kg (±4.26 meq O/Kg); and at 190 °C: 28.88 meq O/Kg (±6.29 meq O/Kg)). While in the frying oil samples with polyphenols, a peroxide concentration of 4.66 meq O/Kg (±1.15 meq O/Kg) was determined, which was statistically equal at all study temperatures (*p* > 0.05) and is also below the maximum limit established in the standards (10 meq O/Kg). The above suggests that the presence of polyphenols allowed the oils to have greater stability against increases in temperature (i.e., in an interval between 145 °C and 190 °C) and, although a certain degree of oxidation was reflected, it was not enough to alter their lipid quality.

The antioxidant effectiveness of methanolic extract of pomegranate husk in samples of sunflower, soybean, and corn oils was previously reported, using concentrations of 0.04% and 0.06% of extract on a dry basis [16]. This evaluation was carried out under accelerated oxidation conditions (70 °C for 10 days) and it was concluded that this extract had the capacity to improve the shelf life of the oils compared to the synthetic antioxidant tert-butylhydroquinone (0.02%). Unlike the previous study, in the present work higher concentrations of polyphenols were used to inhibit the oxidation of VCO during frying; it was necessary because the study temperatures were substantially higher than those used by El-Hadary and Taha (2020) [16] for their accelerated shelf-life study.

As could be seen, in the present work, the efficiency of the antioxidant activity of pomegranate husk polyphenols were directly proportional to their concentration (i.e., 0.05% < 0.1% < 0.2%). Also, it was demonstrated that the antioxidant effect of polyphenols added to VCO was statistically equal (*p* > 0.05) at the three temperatures studied (i.e., 145 °C, 160 °C, and 190 °C). Unlike the other reports where pomegranate extract was used, it is worth noting that VCO has a higher polyunsaturated fatty acid content than sunflower, soybean, or corn oils [30]. This could be why a high percentage of the extract was necessary for the present work to inhibit the formation of peroxides during the thermal processes studied.

### 3.3. Using Vegetable Canola Oil with Polyphenols for Frying Potatoes

**Figure 4:**
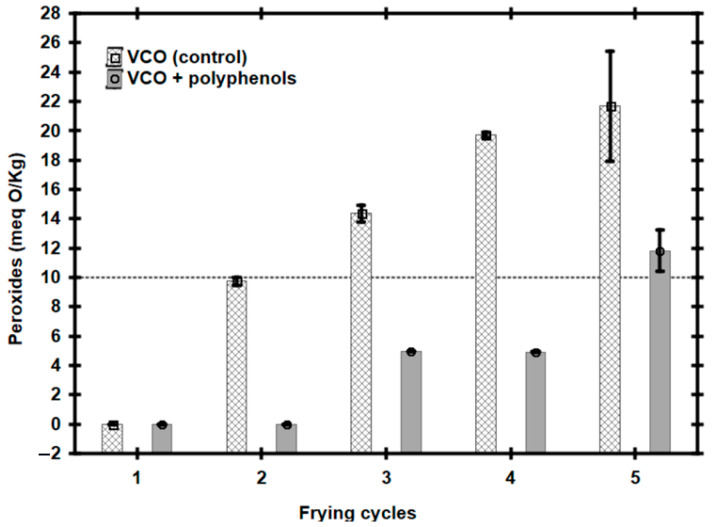
Peroxide concentration in vegetable canola oil (VCO) after each French fries’ preparation frying cycle at 160 °C for 10 min each. The dotted line indicates the specification limit (10 meq O/kg).

The need to economize on the use of vegetable oil to prepare potatoes French fries-style makes its reuse a common practice in Mexico and in many places around the world. Based on the preliminary results of the present work, it was hypothesized that vegetable canola oil (VCO) with polyphenols could be used for this type of frying process. To demonstrate this, the quality of VCO was evaluated by determining the concentration of peroxides after each frying cycle, recycling it for 5 cycles at 160 °C for 10 min (i.e., the time required for the complete cooking of the potatoes in agreement with the instruction label). As a control, a similar process was carried out but using VCO without polyphenols. As was expected, after each frying cycle, the quality of the oil without polyphenols deteriorated because the concentration of peroxides increased with each reuse cycle (Figure 4). At the end of cycle 1, the concentration of peroxides in the sample of VCO was 11.40 meq O/kg (±0.89 meq O/kg) and after cycle 5 it was 22.42 meq O/kg (±2.37 meq O/kg). On the contrary, in the VCO with polyphenols it was observed that it was not until after cycle 5 when the sample presented a concentration of peroxides outside specifications, i.e., 11.27 meq O/kg (±0.89 meq O/kg), and previously in cycle 4 there were 4.92 meq O/Kg (±0.01 meq O/Kg). Previously, in other research work, it had been shown that after heating sunflower oil added with 0.1% of pomegranate extract, in a frying process of 20 cycles at 180 °C, the formation of peroxides was only 10% after 20 frying cycles [31], thus demonstrating that such extract inhibits the formation of peroxides under even higher heat conditions and during more heating cycles than those used in the present work. This confirmed the effectiveness of the antioxidant property of pomegranate extract polyphenols in vegetable oils used in food frying processes. The observed effects could be the result of the ability of the molecules that make up the pomegranate husk polyphenols to act as hydrogen atom donors to lipid radicals, allowing the stability of the systems during heating processes [4,5].

The mechanism of action of antioxidants in these systems will depend on their physicochemical characteristics (i.e., relatively polar, hydrophilic, or amphiphilic antioxidants are more effective in lipid systems, while low-polar or lipophilic antioxidants are more effective in systems with a high surface/volume ratio such as emulsions) and not only on the polar paradox theory [32]. In this case, the polyphenols present in pomegranate husk extract, i.e., punicalagin and granatin B, are partially hydrophilic, but they have proven to be effective antioxidants in systems such as vegetable oils. However, they have the characteristic that they are converted into ellagic acid when hydrolyzed. Ellagic acid is a non-polar phytochemical with higher antioxidant capacity.

So, the polarity of the phytochemicals could be a limitation to the antioxidant effect since the oil–phytochemical interaction is limited. Hence, developing extraction strategies and changing the physicochemical characteristics of phytochemicals, could be helpful to increase their antioxidant effect.

In the present work, it was observed that during frying the polyphenols addition significantly increased the thermal stability of the VCO. Previously, this effect between similar intervals of thermal treatment was also demonstrated by Fukasawa et al. (2009) [33] who reported that polyphenols extracted from rooibos tea protected soybean oil heated to 120–140 °C from oxidation and showed greater effectiveness in processes at 160 °C and 180 °C. Both the results of our work and those of others represents an additional advantage over synthetic antioxidants recommended as frying oil stabilizers, i.e., BHT, BHA, and tocopherols, and is mainly attributed to the fact that polyphenols are much less volatile than those of conventional oils [34]. In the present work, only the lipid peroxidation of the VCO used in the frying process was determined as a first approximation for the identification of the deterioration of the lipids that compose it. This is since, from this peroxidation reaction, a chain of deterioration reactions is initiated, caused by the free radicals that are formed and that mainly attack the unsaturated fatty acids with multiple double bonds and the methylene groups with reactive hydrogen atoms, thus initiating the peroxidation chain. Thus, it has been proposed that with the addition of pomegranate husk polyphenols, the radical scavengers necessary to extinguish the peroxide radicals to end the chain reaction could be provided.

**Figure 5:**
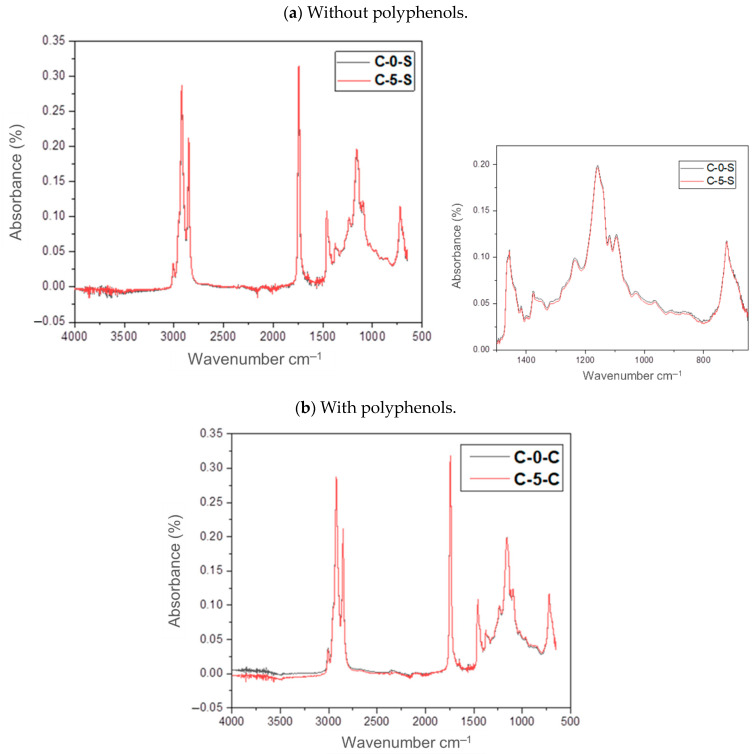
Infrared spectra identified in vegetable canola oil samples without (**a**) and with polyphenols (**b**), after 0 and 5 frying cycles (C-0-C and C-5-C, respectively).

In order to observe the effects at the molecular level of the components of VCO due to the addition of pomegranate husk polyphenols, oil samples without and with polyphenols were analyzed, after 0 and 5 heating cycles, using infrared spectroscopy (Figure 5). In the resulting spectra the only difference was in the oil samples without polyphenols, between cycles 0 and 5. In these samples, the intensity of the peak at 722 cm^−1^ decreased. It was because there was a loss of *cis* double bonds, while the increase in *trans* double bonds increased during thermal oxidation, with greater intensity observed in the peak at 967 cm^−1^ (Figure 5a shortcut). Likewise, the peak at 3008 cm^−1^ was reduced because of the decrease in *cis* allylic bonds (C=CH). In the sample with polyphenols, however, no such changes were identified, i.e., there was no modification of the vibrations of the functional groups because of the application of temperature during the five frying cycles. Additionally, in both samples (Figure 5a,b), the formation of peaks around the 3400 cm^−1^ band was not observed, which corresponds to the vibration of the hydroxyl group (-OH) that can be formed during thermal oxidation, i.e., there was no formation of hydroperoxides. The other bands that were identified, both in Figure 5a,b, are characteristic of the vibrations of the functional groups that constitute the fatty acid chains: the band observed at 1150 cm^−1^ is attributed to C-O stretching; the most prominent absorption band was at 1744 cm^−1^ which is attributed to the C=O stretching of the carbonyl ester functional group of triglycerides. The next most prominent peaks were in the bands at 2922 cm^−1^ and 2852 cm^−1^. These vibrations come from C-H stretching, observing an asymmetric and symmetric stretching of the methylene group (-CH_2_).

## 4. Conclusions

The acetone extraction method and the concentration of polyphenols by chromatography permitted an increase of 5% compared to other methods reported. It was confirmed that the antioxidant properties of polyphenols extracted from pomegranate husk extract were mainly attributed to the ellagitannins it contains, primarily punicalagin, punicalin, and granatin B.

The efficiency of the antioxidant activity of pomegranate husk polyphenols in the vegetable canola oil (VCO), used for frying, was directly proportional to their increase in concentration (i.e., 0.05% < 0.1% < 0.2%). So, it was concluded that the addition of such polyphenols to the VCO allowed it to have greater stability against temperature increases (i.e., in an interval between 145 °C and 190 °C) and, although a certain degree of peroxidation was reflected, it was not enough to alter their lipid quality, remaining under the limits established by regulations (10 meq O/Kg). Also, it was demonstrated that the antioxidant effect of polyphenols added to VCO was statistically equal (*p* > 0.05) at the three temperatures studied (i.e., 145 °C, 160 °C, and 190 °C). Additionally, it was found that the VCO with 0.1% polyphenols can be reused to prepare French fries until four cycles, remaining the peroxide content under the limits permitted (4.92 meq O/Kg (±0.01 meq O/Kg).

Thus, it has been proposed that with the addition of pomegranate husk polyphenols, the radical scavengers necessary to extinguish the peroxide radicals to end the lipid deterioration chain reaction in VCO used for frying processes could be provided. It is suggested that pomegranate husk polyphenols could be an alternative to enhance the shelf life (i.e., cycles of use) of vegetable canola oil used in frying processes in home kitchens, restaurants, and others. In addition, it represents an opportunity to continue investigating the use of such polyphenols for other frying industrial processes.

## Figures and Tables

**Table 1:** Characterization of pomegranate husk polyphenols by HPLC-ESI-MS.

Peak No.	Tentative Identification	RT (min)	Wavelength	[M−H]^−^ *m*/*z*	MS^2^ Ion Fragment (*m*/*z*)
1	Galloyl-hex	7.21	273, 380	331	169, 271
2	Di(HHDP-galloylglucose)-pentose	8.89	278, 380	707 *	613, 633, 461, 635
3	Pedunculagin I	9.46	260, 388	783	481, 301
4	Punicalin	12.20	285, 388	781	301, 479, 601, 723
5	Punicalagin	26.85	280, 388	1083	781, 601, 575
6	Ellagic acid der	28.03	258, 380	799	301, 479
7	Sanguiin-H6	29.07	234, 320	934 *	801
8	Pedunculagin II	30.29	254, 355	785	633, 483, 301, 765
9	Galloyl-HHDP-hex	31.11	262, 373	633	301, 435, 463
10	Granatin B	32.45	266, 370	951	301, 587, 613, 896
11	Ellagic acid	33.79	254, 373	301	185, 229, 257, 272, 284, 301

RT = retention time; * doubly charged.

## Data Availability

The original contributions presented in this study are included in the article. Further inquiries can be directed to the corresponding authors.
